# Kinetics of SuPAR hemoadsorption in critical COVID-19 patients on renal replacement therapy

**DOI:** 10.1186/s12882-022-03003-2

**Published:** 2022-11-18

**Authors:** Vaidas Vicka, Elija Januskeviciute, Ieva Bartuševiciene, Donata Ringaitiene, Aiste Aleknaviciene, Mindaugas Serpytis, Laurynas Rimsevicius, Marius Miglinas, Ligita Jancoriene, Jurate Sipylaite

**Affiliations:** 1 Clinic of Anaesthesiology and Intensive Care, Institute of Clinical Medicine, Faculty of Medicine, Vilnius University, M.K. Ciurlionio 21, 03101 Vilnius, Lithuania; 2 Faculty of Medicine, Vilnius University, Vilnius, Lithuania; 3 Clinic of Gastroenterology, Nephro-urology and surgery, Institute of Clinical Medicine, Faculty of Medicine, Vilnius University, Vilnius, Lithuania; 4 Clinic of Infectious Diseases and Dermatovenerology, Institute of Clinical Medicine, Faculty of Medicine, Vilnius University, Vilnius, Lithuania

**Keywords:** Renal replacement therapy, SuPAR, Hemoadsrobtion

## Abstract

**Background:**

SARS-CoV-2 viral infection is associated with a rapid and vigorous systemic inflammatory response syndrome. Soluble urokinase-type plasminogen activator receptor (suPAR) is a novel biomarker, both indicative of inflammation and propagating it. Hemoadsorption has been proposed as a potential therapy in COVID-19 patients, therefore the aim of this study is to determine suPAR kinetics during hemoadsoprtion.

**Methods:**

This was a prospective observational study of critical COVID-19 patients, enrolled when hemoperfusion therapy was initiated. Hemoadsorber was integrated into the continuous renal replacement therapy circuit. The first series of suPAR measurements was performed 10 minutes after the start of the session, sampling both incoming and outgoing lines of the adsorber. A second series of the measurements was performed beforefinishing the session with the same adsorber. Statistical significance level was set < 0.05.

**Results:**

This study included 18 patients. In the beginning of the session the fraction of suPAR cleared across the adsorber was 29.5% [16-41], and in the end of the session it decreased to 7.2% [4-22], 4 times lower, *p* = 0.003. The median length of session was 21 hours, with minimal duration of 16 hours and maximal duration of 24 hours. The median suPAR before the procedure was 8.71 [7.18-10.78] and after the session was 7.35 [6.53-11.28] ng/ml. There was no statistically significant difference in suPAR concentrations before and after the session (*p* = 0.831).

**Conclusions:**

This study concluded that in the beginning of the hemoadsorption procedure significant amount of suPAR is removed from the circulation. However, in the end of the procedure there is a substantial drop in adsorbed capacity. Furthermore, despite a substantial amount of suPAR cleared there is no significant difference in systemic suPAR concentrations before and after the hemoadsorption procedure.

## Background

SARS-CoV-2 viral infection is associated with a rapid and vigorous systemic inflammatory response syndrome. Innate and adaptive immunity responses may take up a month to fully mature, in some cases being harmful in the development process [1–3]. These responses are associated with an increase in pro-inflammatory cytokines, sometimes causing a deranged response, i.e. “cytokine storm”, which has been shown as a potential factor worsening the clinical outcome [4–6]. One of the treatments advocated to regulate the hyper-inflammatory response is hemoadsorption [7–9]. This blood purification technique is most often carried out by integrating the adsorber in the extracorporeal circuit. It has been reported that levels of various pro-inflammatory cytokines are diminished during the first 24 hours of the therapy [9].

One of the novel inflammatory biomarkers is suPAR, soluble urokinase-type plasminogen activator receptor. This molecule is expressed in the membranes of various immune cells, associated both with innate and adaptive immunity: endothelial cells, macrophages, neutrophils and activated T-cells. During the immune response, this molecule is released into the circulation, showing either an ongoing chronic inflammation or an acute process and may be measured and evaluated in a quantitative manner [10, 11]. SuPAR has been recently reported as the predictor of the acute kidney injury in COVID-19 patients [12]. This biomarker has been described before as a potential starting mechanism of the acute kidney injury, being not only indicative of the damage, but also the cause of it. Furthermore, higher levels of suPAR have been associated with higher admission rates to the ICU, mechanical ventilation and prolonged stay in the hospital [13, 14]. In these studies, the suPAR is reported as a pro-inflammatory cytokine, propagating the deranged immune response [15–17].

There are no clinical trials conducted concerning the hemoadsorption of suPAR. One of the reasons is the size of the suPAR molecule, which is measured from 20 to 50 kDa, depending on the degree of glycosylation and proteolytic cleavage form its membrane form. Therefore, it is unclear to what extent suPAR can be removed during the hemoadsorption procedure. There is a case report, showing that suPAR levels can be diminished up to 27% during the hemoadsorption procedure [18]. However, it is unclear whether this clearance can be sustained during the whole procedure and produce a substantial drop in systemic suPAR concentration. Therefore, the aim of this study is to determine the kinetics of suPAR clearance during hemoadsorption in COVID-19 patients.

## Methods

### Study population

This was a prospective observational study. Sample size of the study was determined from the variability of the suPAR in the Azam et al. study - the median suPAR level reported in the entire cohort was 5.61 ng/ml (IQR, 4.00–7.88), suggesting a SD of less than 1.5 [12]. Therefore, 18 patients were enrolled in the study.

The study was conducted in 2020 autumn-winter wave of COVID-19 patients in Lithuania, that correlates to the 2nd wave in Europe. Selection criteria were: COVID-19 diagnosis, admission to ICU, start of cytokine adsorption. The criteria of initiation of cytokine adsorption were based both on clinical state of the patients, evaluated by the senior physician (oxygen requirements, severity and progression speed, etc.) and by laboratory data indicative of acute systemic response (IL-6 concentration above 100 pg/ml and ferritin concentration above 1000 mcg/L). Exclusion criteria were age < 18y and chronic kidney disease. Study lasted 6-months until 18 consecutive patients were enrolled.

### SuPAR measurements

SuPAR was measured using the The suPARnostic® TurbiLatex (ViroGates A/S, Birkerød, Denmark) test, based on latex particle-enhanced turbidimetric immunoassay that quantitatively determines the suPAR level in human EDTA- or Heparin plasma samples.

The first series of suPAR measurements were performed 10 minutes after start of the perfusion, sampling both incoming and outgoing lines of the adsorber, via the special sampling ports. A second series of the measurements was performed before finishing the first hemoadsorption session with the same adsorber.

### Hemoadsorbtion procedure

Hemoadsorption procedure was performed with a “Cytosorb®” (CytoSorbents Europe GmbH) adsorber, according to the recommendations of the manufacturer. The adsorber was integrated into the continuous renal replacement therapy machine (Fresenius Medical Care multiFiltratePRO Ci-Ca®) before the hemofilter, the modality chosen was continuous veno-venous hemodialysis (CVVHD, filter - Ultraflux AV1000S), with a dialysate flow of 2000 ml per hour, blood flow of 100-150 ml per minute and ultrafiltration according to the clinical state of the patient, ranging from 0 ml per hour to 200 ml per hour, generating a filtration fraction of less than 3%. The length of the procedure planned was 24 hours per one adsorber. The exposure to CRRT was weighted against the possible positive effect of the cytokine adsorption before the decision to initiate the therapy, keeping the best interest of the patient in mind.

### Statistical analysis

Statistical analysis was carried out by the SPSS statistical software package version 26.0 (IBM/SPSS, Inc., Chicago, IL). Baseline characteristics were defined using descriptive statistics. Categorical variables were stated as an absolute number (n) and a relative frequency (%), and continuous variables were represented as a median (interquartile range) or as a mean (± SD), depending on the normality of the distribution. The normality of distribution was tested by one sample Kolmogorov-Smirnov test.

To compare related non-parametric variables Wilcoxon test was used. Linear regression analysis was used to determine the effect of hemadsorption hours on the fraction cleared during the session. Statistical significance level set was < 0.05.

## Results

### Study population

This study included 18 patients treated in the critical care department because of the COVID-19 infection. Baseline characteristics are presented in the Table 1.

**Table 1:** Baseline characteristics of the patients

Demographics	M [IQR] or n (%)
Age, years	54.78 [23-78]
Gender	Male 11 (61.1%)
	Female 7 (38.9%)
**Co-morbidities**	
Obesity	5 (27.8%)
Hypertension	13 (72.2%)
Chronic cardiac disease	3 (16.7%)
Diabetes	4 (22.2%)
**Risk stratification**	
APACHE II	11.11 [4-22]
SOFA	4.44 [2-14]
SAPS II	24.67 [8-45]
4C ISARIC	9.06 [4-14]
**Clinical course**	
Need of MV	13 (76.5%)
Length of MV (days)	9 [6-14.75]
AKI	7 (41.2%)
Need of RRT	6 (35.3%)
Days of symptoms	8 [5.5-11]
Days before ICU	1 [1-2]
LOS in ICU (days)	11 [7-15.25]
LOS in hospital after ICU (days)	27.5 [14.5-33.75]
Mortality	7 (38.9%)

*MV* mechanical ventilation, *AKI* acute kidney injury, *ICU* intensive care unit, *SAPS* Simplified Acute Physiology Score, *SOFA* Sequential Organ Failure Assessment, *APACHE II* Acute Physiology and Chronic Health Evaluation, *4C Mortality ISARIC* 4C International Severe Acute Respiratory and Emerging Infection Consortium Mortality score, *RRT* renal replacement therapy, *LOS* lenght of stay

### Clearance of suPAR across the adsorber

**Fig. 1:**
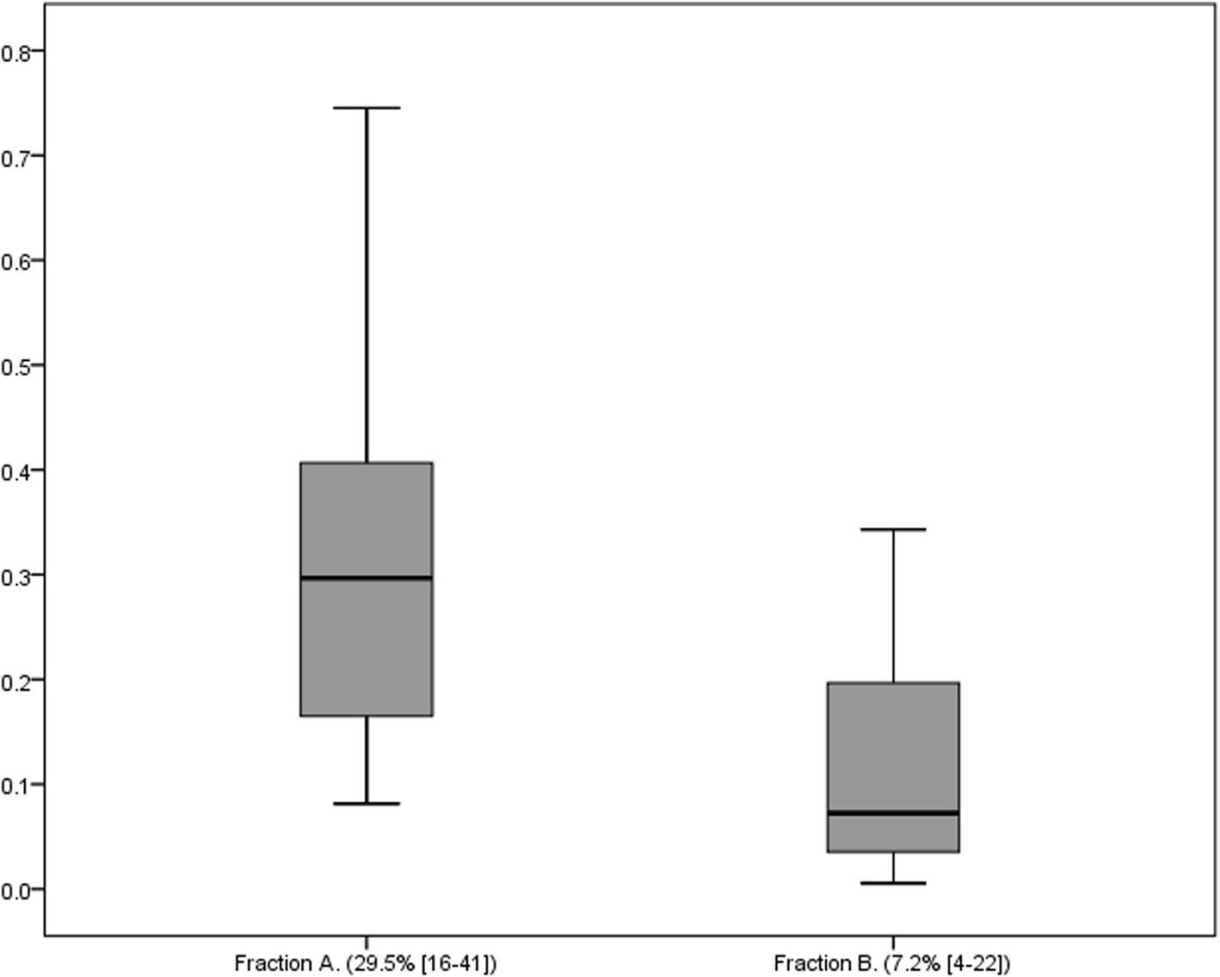
Fraction of suPAR cleared in the beginning and end of the session. Clearence of suPAR in the beginning of the session (Fraction A) and in the end of the session (Fraction B). *P* value = 0.003. X axis denotes the groups, Y axis denotes the fraction cleared

In the beginning of the session the fraction of suPAR cleared across the adsorber was 29.5% [16-41], and in the end of the session it decreased to 7.2% [4-22], 4 times lower, *p* = 0.003. (Fig. 1). After performing the linear regression analysis the tendency of the decrease in adsorber capacity was related to the length of the procedure, per 1 hour decreasing the fraction cleared by 2.4%. (B = 2.4 CI95% 2.0-4.5 *p* = 0.035).

### Clearance of suPAR during the procedure

SuPAR measurements in the beginning of the session were successfully collected for all the patients. In the end of the session only 17 measurements were collected due to hemolysis in the vials for one of the patients. The median length of session was 21 hours, with minimal duration of 16 hours and maximal duration of 24 hours. The most common reason for termination of first session was thrombosis of the circuit 58.8% (*n* = 10), other reasons 11.8% (*n* = 2), 35.5% (*n* = 6) patients successfully finished the 1st session. The median suPAR before procedure measured was 8.71 [7.18-10.78] and after the session was 7.35 [6.53-11.28]. There was no statistically significant difference in suPAR concentrations before and after the session (*p* = 0.831). The measurements of suPAR are presented in Table 2. The overall number of sessions was 3 [2.5-3].

**Table 2:** Clearence of suPAR per session

Changes per session				
	C_IN THE BEGINNING OF THE SESSION_	C_AFTER THE SESSION_	*P* value	C_CHANGE PER SESSION_
Value	8.71 [7.2-10.8]	7.35 [6.5-11.3]	0.831	0.41 [1.0-1.0]
Change in %				5.6 [6.9-15.3]

## Discussion

One of the main findings of our study is that suPAR molecule can be effectively removed with hemoadsorber. In the beginning of the session almost 30% of the incoming suPAR was removed, these results are concordant to the clearance reported in other studies [18]. Also, the degree of clearance reported in our study is similar to other the molecules, which are comparable in size, polarity and ionization [19]. In the ending of the session where was a significant drop of adsorber capacity, plummeting to 7.2%. There is a number of reasons why this decrease could have happened, both conventional and specific to our study. Conventional reasons mainly comprise of saturation of the adsorber membranes, clotting and clogging [20, 21]. Reasons specific to our study are SARS-CoV-2 induced coagulopathy, microthrombosis and length of the procedure [22]. To express the importance of the procedure length, linear regression analysis was performed, linking hours of hemoadsorption with capacity of the adsorbed, showing that clearance capacity decreased by 2.4% per every hour. However, since increment of the regression curve is extrapolated from the samples taken after the 16 hours of the procedure, we cannot say whether there was some sort of non-linear drop of capacity during the first hours of the procedure. All these assumptions and results should be taken into consideration, in this particular time generating more questions rather than answers.

Second finding in our study was no change in systemic suPAR concentration in the beginning and ending of the session. The overall suPAR concentration was rather high in the beginning of the hemoadsorbtion procedure, 8.71 ng/ml. Comparing to the studies of COVID-19 patients the concentration measured in our study was high, indicating an active inflammatory process [12]. However, there are no studies showing suPAR concentrations in COVID-19 patients treated in the ICU, therefore we can not compare our results. After the first session the concentration decreased slightly, only about 5.6%, with no statistical significance, which is comparable to other studies dealing with hemoadsorption. However, there are some insights to be considered. Firstly, the blood flow during extracorporeal circuit in our study was selected to be 100 - 150 ml per minute, providing a high volume of liters perfused via adsorber per day. Adding an average clearance of 5.6% per hour form out study it would let us assume that if suPAR would not be produced during the session it would have been cleared to the minimum. Therefore, even though the overall concentration of suPAR is not being reduced, we can evidently state that a substantial amount is cleared. On the other hand, the concentration of 7.35 ng/ml after the session is still substantially increased, propagating the SIRS and organ failure. This would be expected, since most of the patients in our study were enrolled during their second week of COVID-19 disease, with an ongoing SIRS [23].

There are some limitations to our study. Our results demonstrate the kinetics of suPAR molecule during one hemoadsorption session. It is evident that adsorbers can clear the circulating suPAR molecules effectively, but clinical implementations of these findings are scarce. Firstly, the rate of suPAR production during SIRS, especially in COVID-19 patients is unknow. Form our study we can see that despite clearing a substantial amount of suPAR we did not achieve a lower concertation, suggesting an ongoing active production, which negates the amount cleared. Secondly, the measurements and sampling in our study were done in cross-sectional manner in the beginning and ending of the session, creating a static model, stagnant in representing real clinical cases. Furthermore, the sample size was small, complicating the statistical analysis and leaving the assumptions what would have been if large sample was enrolled. Finally, it is still unknown if stabilizing or lowering the concentration of suPAR would help to achieve better clinical outcomes.

## Conclusions

This study concluded that in the beginning of the hemoadsorption procedure significant amount of suPAR is removed from the circulation. However, in the end of the procedure there is a substantial drop in adsorbed capacity. Furthermore, despite a substantial amount of suPAR cleared there is no significant difference in systemic suPAR concentrations before and after the hemoadsorption procedure.
